# Metabolomics-based profiles predictive of low bone mass in menopausal women

**DOI:** 10.1016/j.bonr.2018.06.004

**Published:** 2018-06-18

**Authors:** Takeshi Miyamoto, Akiyoshi Hirayama, Yuiko Sato, Tami Koboyashi, Eri Katsuyama, Hiroya Kanagawa, Atsuhiro Fujie, Mayu Morita, Ryuichi Watanabe, Toshimi Tando, Kana Miyamoto, Takashi Tsuji, Atsushi Funayama, Tomoyoshi Soga, Masaru Tomita, Masaya Nakamura, Morio Matsumoto

**Affiliations:** a Department of Orthopedic Surgery, Keio University School of Medicine, 35 Shinano-machi, Shinjuku-ku, Tokyo 160-8582, Japan; b Department of Advanced Therapy for Musculoskeletal Disorders, Keio University School of Medicine, 35 Shinano-machi, Shinjuku-ku, Tokyo 160-8582, Japan; c Department of Musculoskeletal Reconstruction and Regeneration Surgery, Keio University School of Medicine, 35 Shinano-machi, Shinjuku-ku, Tokyo 160-8582, Japan; d Department of Dentistry and Oral Surgery, Keio University School of Medicine, 35 Shinano-machi, Shinjuku-ku, Tokyo 160-8582, Japan; e Institute for Advanced Biosciences, Keio University, 246-2 Mizukami, Kakuganji, Tsuruoka, Yamagata 997-0052, Japan

**Keywords:** Metabolomics, Metabolite, Women, Estradiol, Estrogen, Menopause, Bone mineral density

## Abstract

Osteoporosis is a skeletal disorder characterized by compromised bone strength and increased risk of fracture. Low bone mass and/or pre-existing bone fragility fractures serve as diagnostic criteria in deciding when to start medication for osteoporosis. Although osteoporosis is a metabolic disorder, metabolic markers to predict reduced bone mass are unknown. Here, we show serum metabolomics profiles of women grouped as pre-menopausal with normal bone mineral density (BMD) (normal estrogen and normal BMD; NN), post-menopausal with normal BMD (low estrogen and normal BMD; LN) or post-menopausal with low BMD (low estrogen and low BMD; LL) using comprehensive metabolomics analysis. To do so, we enrolled healthy volunteer and osteoporosis patient female subjects, surveyed them with a questionnaire, measured their BMD, and then undertook a comprehensive metabolomics analysis of sera of the three groups named above. We identified 24 metabolites whose levels differed significantly between NN/LN and NN/LL groups, as well as 18 or 10 metabolites whose levels differed significantly between NN/LN and LN/LL, or LN/LL and NN/LN groups, respectively. Our data shows metabolomics changes represent useful markers to predict estrogen deficiency and/or bone loss.

## Highlights

• Comprehensive metabolomic analysis was undertaken in women with normal or low estradiol and normal or low bone mass.
• 52 metabolites were identified as biomarkers of estrogen deficiency and/or low bone mass.
• Metabolomic profiling could predict estrogen-deficiency and/or low bone mineral density in postmenopausal women.

## 1 Introduction

Osteoporosis is characterized by risk of bone fragility fracture due to several mechanisms, among them, low bone mass owing to aging and hypogonadism (Assessment of fracture risk and its application to screening for postmenopausal osteoporosis, 1994; NIH Consens. Statement, 2000). The number of osteoporosis patients is currently increasing in developed countries worldwide due to aging populations (Reginster and Burlet, 2006). To prevent fragility fractures, patients suspected of having osteoporosis are diagnosed and treated with anti-osteoporosis drugs (Marshall et al., 1996). The criteria for treatment includes bone mineral density (BMD) lower than −2.5SD and fragility fractures in vertebra or hips (Das and Crockett, 2013; Kanis et al., 2013; Siris et al., 2014). Moreover, a fracture risk assessment tool (FRAX) can assess future fragility fracture risks and is now utilized to diagnose patients needing treatment or to avoid treating patients unnecessarily (Kanis et al., 2008). Recent cohort studies reveal that more than half of females over 80 years old in the general population have been diagnosed with osteoporosis based on lower BMD values (Yoshimura et al., 2009), suggesting that more than half of all women become osteoporotic with aging as a natural course of events. Elderly females diagnosed with osteoporosis based on a BMD value lower than −2.5SD are estimated show a bone mass 70% of the young adult mean (YAM); however, increases in BMD following osteoporosis therapy are reportedly limited to 7–10% or 3–5% of baseline BMD in the lumbar spine or femoral neck, respectively (Black et al., 1996; Cummings et al., 2009; Ettinger et al., 1999; Harris et al., 1999). Thus, blocking future loss from the peak bone mass would likely be a more effective way to prevent bone fragility fractures correlated with low bone mass. Doing so requires predictive tools, which are currently unavailable.

Recently, changes in levels of metabolites have been associated with altered bone mineral density in human (You et al., 2014; Qi et al., 2016; Miyamoto et al., 2017; Moayyeri et al., 2017) or animal models (Lee et al., 2016; Huang et al., 2016; Ma et al., 2013), or in cultured osteoclastic cells in vitro (Liu et al., 2015). However, comprehensive, systemic metabolomics studies in humans aimed at identifying candidate metabolites associated with low bone mineral density or estrogen deficiency have been limited, requiring further studies to understand menopausal changes in particular. Previously, we used comprehensive metabolomic analysis to show that blood levels of the di-peptides Gly-Gly and Cystine are significantly lower in sera of post-menopausal females exhibiting low relative to normal BMD (Miyamoto et al., 2017). We concluded that both metabolites could be useful markers to predict low BMD without BMD measurements, which require dual energy X ray absorptiometry (DEXA) and are costly (Miyamoto et al., 2017). Here, we searched for additional candidate metabolites useful as markers of low BMD.

Here, we newly recruited female subjects, including pre- and post-menopausal women, and subdivided them into three groups: 1, pre-menopausal and normal BMD (with normal estrogen and normal BMD, NN); 2, post-menopausal and normal BMD (with low estrogen and normal BMD, LN) and 3, post-menopausal and low BMD (with low estrogen and low BMD, LL). We then collected sera for comprehensive metabolomics analysis. Gly-Gly and Cystine levels were significantly lower in LL than in LN groups, confirming our previous study. We also identified 10 metabolites whose levels differed significantly between LN and LL groups. We also identified 24 and 18 metabolites whose levels differed significantly between NN and LN, and LN and LL groups, respectively. Overall, we conclude that our metabolomics profiles could serve as a useful diagnostic tool to monitor the metabolic changes accompanying estrogen deficiency and subsequent bone loss.

## 2 Materials and methods

### 2.1 Subjects

Subjects were female employees of the Keio University School of Medicine, aged 39–61 years, who had undergone medical examination in September of 2013 (Miyamoto et al., 2017; Miyamoto et al., 2016) or who had visited our hospital as possible patients. Written informed consent was obtained from all subjects. All subjects were Japanese. Each completed a self-reported questionnaire regarding menopausal status and drug usage. This study was approved by an ethics committee at Keio University School of Medicine and carried out in accordance with guidelines approved by that committee.

### 2.2 Measurements

All subjects were asked to fast overnight and were assessed for height, body weight, serum calcium (Ca), inorganic phosphorus (IP), 25(OH)D, intact parathyroid hormone (PTH), TRACP5b and estradiol (E2) levels. Body mass index (BMI) was calculated from body weight and height data. Serum 25(OH)D and intact serum PTH levels were analyzed by using an 125I RIA kit (DiaSorin, Stillwater, MN, USA) and an ECLIA kit (Cobas, Roche Diagnostics, Basel, Switzerland), respectively. Bone mineral density (BMD) was analyzed in all subjects using an AOS-100 system (Aloka, Tokyo, Japan) or dual-energy X-ray absorptiometry (DEXA; GE Healthcare, Amersham Place, Little Chalfont, Buckinghamshire HP7 9NA, England).

Statistical analysis was performed using the unpaired two-tailed Student's or Welch's *t*-test (*, *p* < 0.05; **, *p* < 0.01; ***, *p* < 0.001; NS, not significant). All data are shown as means ± S.D.

### 2.3 Metabolite extraction from serum

Extraction was performed as described (Miyamoto et al., 2017). Briefly, 40 μl aliquots of serum from subjects were mixed with 360 μl methanol containing internal standards (20 μmol/l each of methionine sulfone and D-camphor-10-sulfonic acid). Solutions were shaken with 400 μl chloroform and 160 μl Milli-Q water, and then centrifuged at 10,000 ×*g* for 3 min at 4 °C. The aqueous layer was removed and filtered using a 5-kDa-cutoff filter (Human Metabolome Technologies, Tsuruoka, Japan) to remove protein. The filtrate was dried using a centrifuge concentrator and reconstituted in 50 μl of Milli-Q water containing reference compounds (200 μmol/l each of 3-aminopyrrolidine and trimesic acid) prior to CE-TOFMS analysis.

.

### 2.4 CE-TOFMS metabolome analysis

All CE-TOFMS analyses were performed using an Agilent 1600 Capillary Electrophoresis system (Agilent Technologies, Waldbronn, Germany) equipped with an Agilent 6210 TOF LC/MS system (Agilent Technologies, Santa Clara, CA) as described (Soga et al., 2009). For anionic metabolite analysis, the ESI sprayer was replaced with a platinum rather than a stainless steel needle as described (Soga and Heiger, 2000). Cationic metabolites were separated through a fused-silica capillary (50 μm i.d. × 100 cm) filled with 1 mol/l formic acid as electrolyte (Hirayama et al., 2015), and then a methanol/water (1:1) solution containing 0.1 μmol/l hexakis(2,2-difluoroethoxy) phosphazene was delivered as sheath liquid at a rate of 10 μl/min. The capillary temperature was maintained at 20 °C.

The sample solution was injected at 5 kPa for 3 s, and the separation voltage was set at 30 kV. ESI-TOFMS was conducted in positive ion mode, and capillary, fragmentor, skimmer and Oct RF voltages were set at 4000, 75, 50 and 125 V, respectively. Nebulizer gas pressure was configured at 7 psig, and heated (300 °C) nitrogen gas was supplied at a rate of 10 L/min. Anionic metabolites were separated through a COSMO(+) capillary (50 μm i.d. × 105 cm, Nacalai Tesque, Kyoto, Japan) filled with 50 mmol/l ammonium acetate (pH 8.5) as electrolyte (Soga et al., 2009), and an ammonium acetate (5 mmol/l) in a (1:1) methanol/water solution containing 0.1 μmol/l hexakis(2,2-difluoroethoxy)phosphazene was delivered as sheath liquid at a rate of 10 μl/min. The sample solution was injected at 5 kPa for 30 s. Negative 30 kV was applied for sample separation. ESI-TOFMS was conducted in negative ion mode, and capillary, fragmentor, skimmer and Oct RF voltages were set at 3500, 100, 50 and 200 V, respectively. Other conditions were identical for cationic metabolite analysis.

In both modes, an automatic recalibration function was used to correct for analytical variation of exact masses for each run, as described (Hirayama et al., 2015). Exact mass data were acquired at a rate of 1.5 cycles/s over a 50–1000 *m*/*z* range.

Raw data were processed using our proprietary automatic integration software (MasterHands) (Sugimoto et al., 2010; Sugimoto et al., 2012). Each peak was identified by matching *m*/*z* values and normalized migration times of corresponding authentic standard compounds.

Statistical analysis was performed using the Mann-Whitney *U* test (*, *p* < 0.06; **, *p* < 0.01; ***, *p* < 0.001; NS, not significant).

## 3 Results and discussion

### 3.1 Basic characteristics of study subjects

**Fig. 1:**
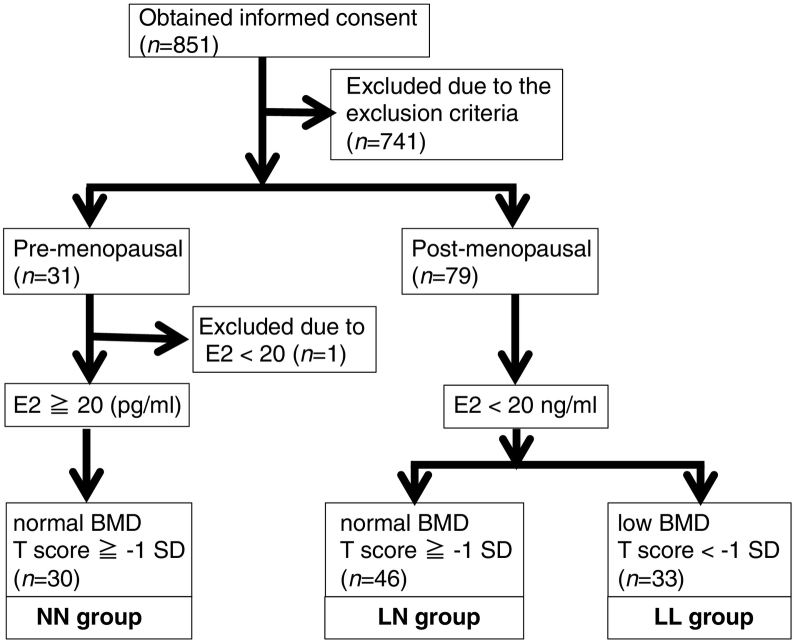
Selection of study subjects. Written informed consent was obtained from 851 women in our hospital, and 741 subjects were excluded based on indicated criteria. Thus, the number of enrolled subjects was: normal estradiol and normal BMD (NN) group, 30; low estradiol with normal BMD (NL) group, 46; and low estradiol with low BMD (LL) group, 33. Exclusion criteria included drug usage (not limited to anti-osteoporosis drugs), unstable menstruation status, lean or obese status (BMI < 18.5 or BMD > 30, respectively) and incomplete data sets. BMI, body mass index; E2, estradiol; BMD, bone mineral density.

We invited 597 women aged 39–64 years, who had undergone a medical examination to participate in this study and obtained informed consent from 526. We also obtained informed consent from 325 patients who had visited our osteoporosis out-patient clinic for the first time. Subjects with the following conditions were excluded (*n* = 741) from the study: those who had used medications including but not limited to osteoporosis drugs; those with unstable menstruation status; subjects exhibiting a BMI of <18.5 or >30; and individuals with incomplete data sets (Fig. 1). Then, the remaining subjects (*n* = 110) were divided into pre- and post-menopausal groups, and the latter was subdivided into “low BMD” (lower than −1 SD (low estradiol [E2] and low BMD; LL) and “normal BMD” (low E2 and normal BMD; LN) groups. One subject in the pre-menopausal group who showed <20 pg/ml E2 was excluded (Fig. 1). Since pre-menopausal individuals exhibited normal bone mass (higher than −1 SD), these individuals were grouped as normal E2 and normal BMD (NN group). The number of subjects in final NN, LL, and LN groups were 30, 46, and33, respectively (Fig. 1).

We observed significant differences between NN and LN groups in values related to age, height, BMI, Ca, TRACP5b, BMD and E2 (Table 1). Values related to age, height, body weight, TRACP5b, BMD and E2 also significantly differed between NN and LL groups, while age, height, body weight, BMI, Ca, 25(OH)D, TRACP5b, BMD and E2 differed significantly between LN and LL groups (Table 1). At present, why the LL group showed such great variation in PTH levels, as reflected by high SDs is not clear. PTH levels are reportedly inversely correlated with those of vitamin D (Miyamoto et al., 2016; Garnero et al., 1996), and inverse correlation between the two was observed in our subjects, including in the LL group (Table 2). Low vitamin D levels, as indicated by 25(OH)D values lower than 30 ng/ml, were seen in all groups (Table 1). Since vitamin D regulates bone homeostasis, we cannot exclude the possibility that low vitamin D levels influence metabolomics profiles. However, 25(OH)D levels were not associated with either TRACP5b or BMD values in any group or in the total population (Table 3). Moreover, low vitamin D levels are often seen in the general population, even in people under 40 years old (Miyamoto et al., 2016), as well as in our subjects. Thus, our metabolomics data may reflect general trends.

**Table 1:** Basic characteristics of the study population.

	Normal E2 & normal BMD (NN group)	Low E2 & normal BMD (LN group)	Low E2 & low BMD (LL group)	*p* value (NN vs LN)	*p* value (NN vs LL)	*p* value (LN vs LL)
Age (years)	41.7 ± 1.5	60.9 ± 9.9	71.3 ± 8.9	***	***	***
Height (cm)	158.3 ± 3.6	154.7 ± 5.9	150.7 ± 8.9	**	***	**
Body weight (kg)	51.1 ± 4.6	53.0 ± 9.4	46.4 ± 7.7	NS	**	***
BMI (kg/m^2^)	20.4 ± 1.7	22.1 ± 3.3	20.5 ± 3.6	**	NS	*
Ca (mg/dl)	9.2 ± 0.3	9.4 ± 0.3	9.3 ± 0.3	**	NS	*
IP (mg/dL)	3.7 ± 0.4	3.7 ± 0.4	3.8 ± 0.4	NS	NS	NS
25(OH)D (ng/ml)	21.5 ± 6.6	22.5 ± 6.0	19.6 ± 8.3	NS	NS	*
Intact PTH (pg/ml)	49.8 ± 12.7	51.8 ± 16.5	61.9 ± 43.7	NS	NS	NS
TRACP5b (mU/dL)	186.6 ± 66.5	385.3 ± 171.3	506.8 ± 184.6	***	***	**
BMD: *t* score (SD)	0.8 ± 0.8	−0.2 ± 0.9	−2.7 ± 0.7	***	***	***
E2 (pg/ml)	174.2 ± 147.9	10.0 ± 0.0	10.1 ± 0.4	***	***	*

**Table 2:** Correlation of serum PTH and 25(OH)D values.

	*r* value	*p* value
NN group	−0.38	*
LN group	−0.082	NS
LL group	−0.31	0.079
Total	−0.25	**

**Table 3:** Association between 25(OH)D and TRACP5b (upper) or BMD (lower) values.

25(OH)D and TRACP5b		
	*r* value	*p* value
NN group	0.086	NS
LN group	−0.164	NS
LL group	−0.189	NS
Total	−0.158	0.098

25(OH)D and BMD		
	*r* value	*p* value
NN group	−0.303	0.097
LN group	0.094	NS
LL group	0.242	NS
Total	−0.086	NS

### 3.2 Candidate metabolites indicative of low estrogen in post-menopausal subjects

We then undertook comprehensive metabolomic analysis of sera from subjects in all groups and identified 102 metabolites using CE-TOFMS.

**Fig. 2:**
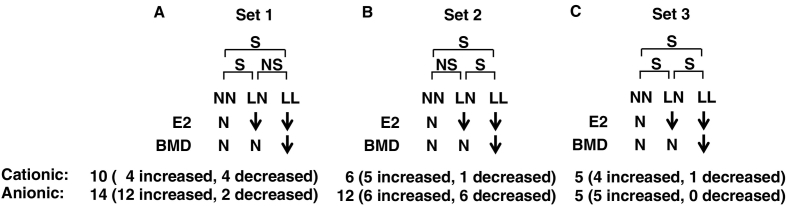
Subject and metabolite profiles. Subjects were subdivided into three groups: group 1, normal estradiol (E2) and normal bone mineral density (BMD) (NN); group 2, low E2 with normal BMD (LN); and group 3, low E2 with low BMD (LL). Metabolites identified were subdivided into three sets: 1, levels differed significantly different NN and LN or between NN and LL groups but were comparable in LN and LL; 2, levels differed significantly between NN and LL, or between LN and LL but were equivalent between NN and LN groups; 3, significantly different levels were seen between NN and LN, LN and LL, and NN and LL groups. S, significant; NS, not significant, N, normal levels. Arrows indicate down-regulation.

We then subdivided metabolites into the following sets. In set 1, metabolite levels differed significantly between NN and LN and between NN and LL groups, but were comparable in LN and LL groups. Estrogen levels differed significantly between NN and LN and between NN and LL groups (Fig. 2). Aging is reportedly a strong confounding factor that can affect levels of various metabolites (Yu et al., 2012; Trabado et al., 2017). Since NN subjects were premenopausal, while LN and LL subjects were postmenopausal, the effects of aging cannot be excluded when interpreting changes in metabolomics profiles between NN and LN or NN and LL groups. In set 2, metabolite levels differed significantly between NN and LL and between LN and LL groups but were similar between NN and LN groups. These outcomes likely reflect BMD differences (Fig. 2). Finally, in set 3, metabolite levels differed significantly between NN and LN, LN and LL, and NN and LL groups. We conclude that these differences reflect differences in estrogen levels, BMD, or both (Fig. 2).

**Fig. 3:**
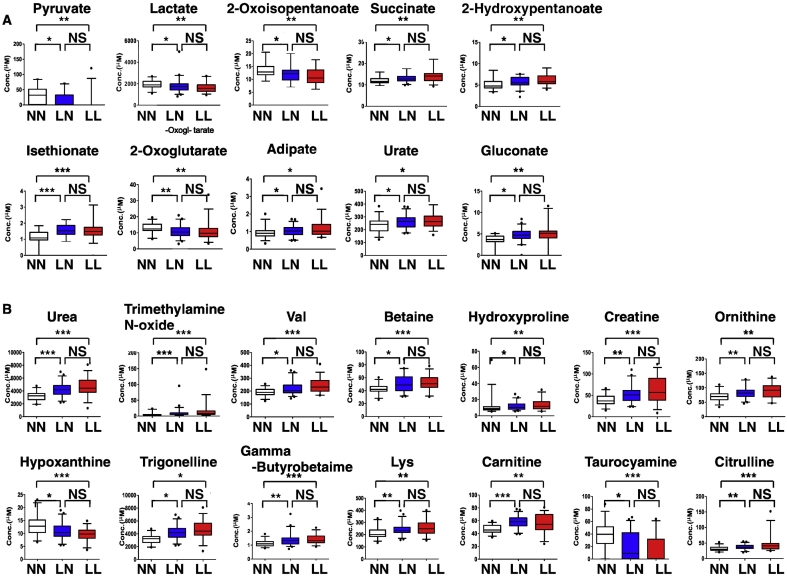
Metabolites significantly changed by estrogen deficiency. Comprehensive metabolomic analysis was performed in sera from NN, LN and LL subjects, and metabolite levels compared between groups. Metabolites showing significant differences between NN and LN or NN and LL groups are shown. (A, cationic; B, anionic). Data represent mean levels of indicated metabolite ± SD (μM; *n* = 30, 46 and 33 for respective NN, LN and LL subjects; *p < 0.05. **p < 0.01).

We identified 10 (6 upregulated and 4 downregulated) cationic and 14 (12 upregulated and 2 downregulated) anionic metabolites in set 1 (Fig. 3).

### 3.3 Candidate metabolites indicative of low BMD in post-menopausal subjects

**Fig. 4:**
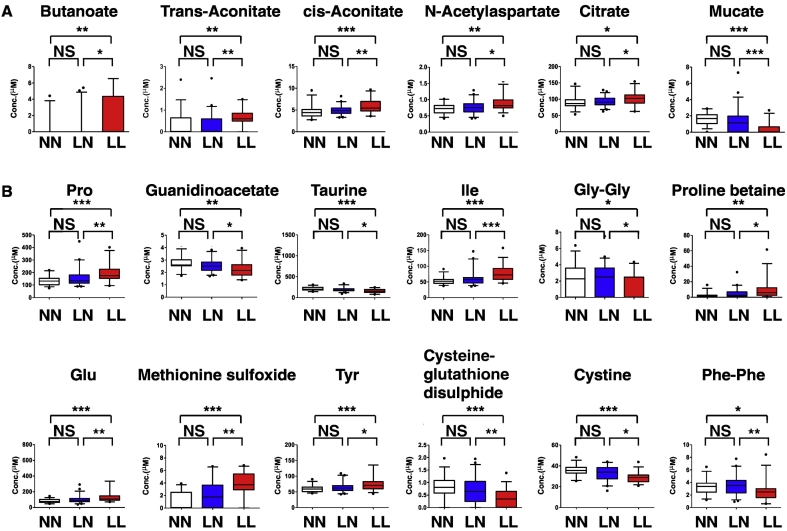
Metabolites significantly changed by low BMD. Metabolite levels differing significantly between NN and LN or NN and LL groups are shown. (A, cationic; B, anionic). Data represent mean levels of indicated metabolite ± SD (μM; n = 30, 46 and 33 for NN, LN and LL subjects; *p < 0.05, **p < 0.01).

As noted above, we defined metabolite set 2 as candidate predictors of low bone mass in post-menopausal women (Fig. 4). We detected 6 (5 upregulated and 1 downregulated) and 12 (6 upregulated and 6 downregulated) cationic and anionic metabolites in this set, respectively. We found that levels of Gly-Gly and Cystine were significantly lower in LL than in LN groups, validating our earlier study (You et al., 2014) (Fig. 4). Vitamin D levels are reportedly associated with variations in metabolite levels (Lasky-Su et al., 2017; Finkelstein et al., 2015; Vogt et al., 2016), and 25(OH)D levels differed significantly between LL and LN groups (Table 1). Moreover, 25(OH)D levels were not associated with BMD in our cohort (*β*, −0.0697; P value, 0.5843). Since Gly-Gly and Cystine levels significantly differed between LN and LL groups, we consider them useful predictors of low bone mass in post-menopausal women.

### 3.4 Candidate metabolites indicative of low estrogen and BMD in post-menopausal subjects

**Fig. 5:**
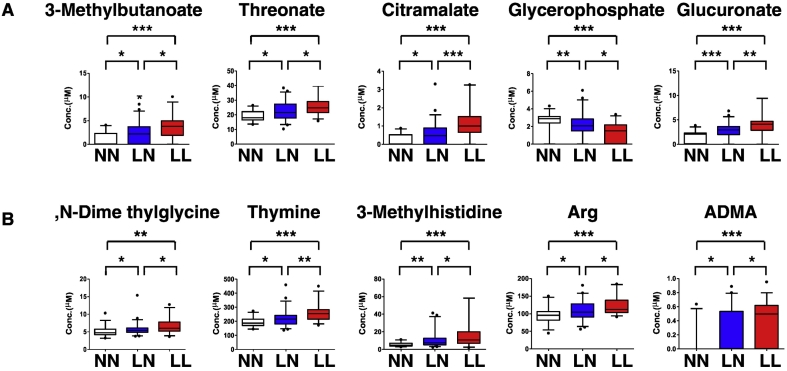
Metabolites significantly changed by estrogen deficiency and low BMD. Metabolites showing significant differences between NN and LN or NN and LL groups are shown. (A, cationic; B, anionic). Data represent mean levels of indicated metabolite ± SD (μM; n = 30, 46 and 33 for NN, LN and LL subjects; *p < 0.05, **p < 0.01).

Finally, we detected 5 cationic and 5 anionic metabolites as metabolite set 3 (Fig. 5). Since these metabolites differed significantly between the LN and LL group, as did set 2, we conclude that they are useful to predict low bone mass in post-menopausal women.

For each metabolite, levels of changes from baseline varied, but a combination of changes of metabolite profiles will help understanding and diagnosis for changes of metabolic status of bones in each individual.

Low bone mass, which is the best-known risk for fragility fractures associated with osteoporosis, is promoted by metabolic changes in bones of elderly, post-menopausal women. Thus, metabolic profiling could reveal markers useful to monitor changes predictive of low bone mass. Our previous metabolomic analysis in post-menopausal women identified low levels of Gly-Gly and Cystine (Miyamoto et al., 2017) as potential markers. Our new analysis confirms significant down-regulation of both in LL than LN groups, levels unchanged between NN and LN groups, suggesting that changes reflect altered post-menopausal bone mass rather than estrogen deficiency. Here, we identified 16 additional candidates as markers of low post-menopausal bone mass, bringing the total to 18 biomarkers diagnostic of low bone mass. Levels of 10 metabolites significantly changed between LN and LL and between NN and LN groups. Although these changes may reflect estrogen deficiency, they could also serve as predictors of low bone mass.

It remains challenging to increase bone mass from lower than −2.5 SD/70% YAM BMD in the elderly. The most widely used bisphosphonate can increase femoral BMD approximately 3% after three years of continuous treatment, and BMD elevation reportedly plateaus after this time period (Cummings et al., 1998). The monoclonal antibody Denosumab is reportedly more effective in elevating femoral BMD, but even three years of continuous Denosumab treatment increases femoral BMD approximately 5% (Cummings et al., 2009), and treatment for a total of eight and ten years elevates femoral BMD approximately 8% and 9%, respectively (Papapoulos et al., 2015; Bone et al., 2017). Teriparatide, also known as parathyroid peptide hormone 1–34, and selective estrogen receptor modulators are less effective in increasing femoral BMD than are bisphosphonates or Denosumab (Ettinger et al., 1999; Itabashi et al., 2011; Eriksen et al., 2014). Therefore, prevention of low BMD based on novel diagnostic tools is a preferable option. We propose that monitoring metabolomic status before and after menopause could sense decreases in BMD, allowing for timely intervention and possible delay of low bone mass status, decreasing the risk for fragility fractures.

**Fig. 6:**
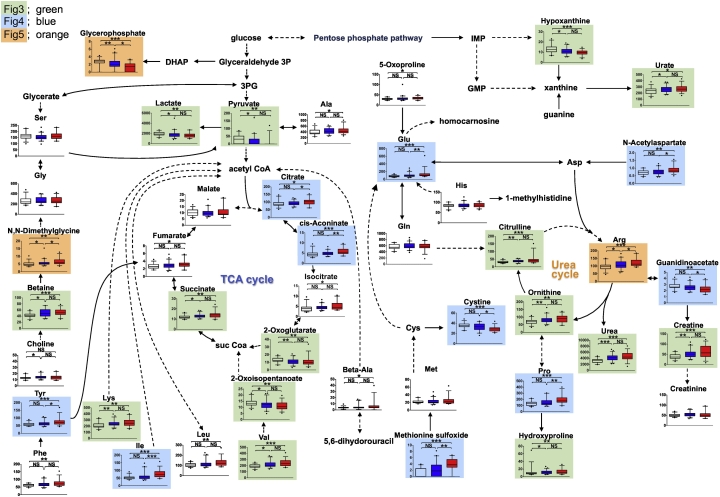
Schematic showing interaction of metabolites. Shown are metabolites functioning in the TCA or urea cycles, and other pathways. Metabolites highlighted by green, blue or orange are analyzed in Fig. 3, Fig. 4, Fig. 5, respectively. (For interpretation of the references to colour in this figure legend, the reader is referred to the web version of this article.)

Fig. 6 shows function of metabolites identified here in various biochemical pathways. Levels of two metabolic intermediates seen early in the tricarboxylic acid (TCA) cycle, citrate and cis-aconitate, increased in the LL group. The TCA cycle is a critical metabolic pathway regulating oxidative conversion of carbohydrates to generate cellular energy, and aconitase is required to metabolize both citrate and cis-aconitate. The activity of aconitase, a member of the iron‑sulfur-containing hydratase family, is highly sensitive to oxidative stress (Sohal, 2002). A recent study reported that rapid BMD decreases seen in most menopausal women might result from decreased defense against oxidative stress (Qi et al., 2016). Taken together, aconitase activity in low BMD patients was suppressed by severe oxidative stress, potentially leading to accumulation of citrate and cis-aconitate in this group.

Hydroxyproline is abundant in collagen and functions in collagen stability. Collagen fibers confer tensile strength to bone, and hydroxyproline is released from bone as collagen degrades. Therefore hydroxyproline content in serum or urine is a marker of osteoclastic bone resorption (Delport et al., 2004). Relative to the NN group, hydroxyproline concentrations significantly increased in post-menopausal (LN and LL) groups in this study. Furthermore, hydroxyproline levels tended to increase in the LL group, although increases were not significant. A previous study reported increased plasma hydroxyproline levels in ovariectomized rats, a widely accepted postmenopausal osteoporosis model, at different time intervals after surgery (Ma et al., 2013). This result is consistent with our observation and supports the idea that hydroxyproline may be a potential biomarker of postmenopausal osteoporosis.

Concentrations of branched chain amino acids (BCAAs), including valine, isoleucine and leucine, significantly increased in the LL group. BCAAs are vital for protein biosynthesis and play an important role in skeletal muscle maintenance and stabilization. For example, BCAAs antagonize muscle breakdown by preventing amino acid loss from muscle (Huang et al., 2016). Also, muscle strength and bone mass are positively correlated (Frost, 1997). In our study, levels of most amino acids, except BCAAs, increased in the LL group. Thus patients with low BMD and decreased muscle strength may show aberrantly high levels in blood of amino acids derived from muscle proteins.

Interestingly, we observed significant differences in the urea cycle and creatine biosynthesis pathway in all groups. The urea cycle is the final pathway to remove surplus nitrogen from the body, and is the primary route for ammonia detoxification in humans (Walker, 2009). Levels of arginine, citrulline, ornithine, urea and creatine increased in post-menopausal groups, while levels of guanidinoacetate decreased. Up to now, there has been no relationship reported between a menopausal state and changes in the urea cycle, and there are several explanations for our findings. First, increased urea cycle flux in post-menopausal groups may occur in response to excess ammonia in blood as a means of detoxification. Second, since we observed an inverse relationship between guanidinoacetate and both arginine and creatine, activities of enzymes catalyzing metabolism of these factors may change. Guanidinoacetate is metabolized to creatine by guanidinoacetate *N*-methyltransferase and reversibly metabolized to arginine by glycine amidinotransferase.

A strength of this study is that can detect changes in broad variety of hydrophilic metabolites that reflect the complexity of metabolic networks altered in estrogen deficiency or low bone mass. However, there are several limitations to be acknowledged for this study. Subjects enrolled were recruited at our hospital, and future studies are required before findings are generalized to subjects in other areas and populations. The number of subjects analyzed was also limited due to exclusion of subjects administered medications including but not limited to osteoporosis drugs. Indeed, we confirmed significantly reduced serum Gly-Gly and Cystine levels in LL relative to LN groups. Nonetheless, a replication group or a future validation study is warranted to confirm our findings relevant to other metabolites. It is currently not possible to predict a future decrease in BMD in postmenopausal women who show normal BMD. Follow-up studies to analyze changes in BMD in each group could identify biomarkers to predict future low bone mass. In addition, follow up studies to detect changes in metabolomics status relative to bone mineral density in subjects before and after the menopause could be useful clinically to predict low bone mass. Furthermore, this study focused only on hydrophilic metabolites. Lipid species, such as fatty acids, diglycerides, triglycerides and cholesterols, are also important metabolomic factors. Thus, use of multiple analytical platforms is needed to expand this analysis. Our study does not identify mechanisms underlying metabolite level changes due to estrogen deficiency or low bone mass, nor do we yet fully understand interactions between metabolites. Moreover, evaluation of metabolomic status could aid diagnosis of individuals with a genetic predisposition or family history of osteoporosis, or alcohol or smoking habits associated with the condition (Kanis et al., 2004; Kanis et al., 2005a; Takeshima et al., 2017; Kanis et al., 2005b; Mito et al., 2017). Nonetheless, our study suggests a novel and reliable diagnostic tool to predict low bone mass.

## Transparency document

Transparency document. Image 1
